# Transcription-Based Multidimensional Regulation of Fatty Acid Metabolism by HIF1α in Renal Tubules

**DOI:** 10.3389/fcell.2021.690079

**Published:** 2021-07-02

**Authors:** Wenju Li, Aiping Duan, Yuexian Xing, Li Xu, Jingping Yang

**Affiliations:** ^1^National Clinical Research Center of Kidney Diseases, Jinling Hospital, Medical School of Nanjing University, Nanjing, China; ^2^Department of Otorhinolaryngology-Head and Neck Surgery, Jinling Hospital, Medical School of Nanjing University, Nanjing, China; ^3^Medical School of Nanjing University, Nanjing, China

**Keywords:** lipid metabolism, HIF, transcription regulation, lipidome, mitochondria

## Abstract

Lipid metabolism plays a basic role in renal physiology, especially in tubules. Hypoxia and hypoxia-induced factor (HIF) activation are common in renal diseases; however, the relationship between HIF and tubular lipid metabolism is poorly understood. Using prolyl hydroxylase inhibitor roxadustat (FG-4592), we verified and further explored the relationship between sustained HIF1α activation and lipid accumulation in cultured tubular cells. A transcriptome and chromatin immunoprecipitation sequencing analysis revealed that HIF1α directly regulates the expression of a number of genes possibly affecting lipid metabolism, including those associated with mitochondrial function. HIF1α activation suppressed fatty acid (FA) mobilization from lipid droplets (LDs) and extracellular FA uptake. Moreover, HIF1α decreased FA oxidation and ATP production. A lipidomics analysis showed that FG-4592 caused strong triglyceride (TG) accumulation and increased some types of phospholipids with polyunsaturated fatty acyl (PUFA) chains, as well as several proinflammatory lipids. Nevertheless, the overall FA level was maintained. Thus, our study indicated that HIF1α reduced the FA supply and utilization and reconstructed the composition of lipids in tubules, which is likely a part of hypoxic adaptation but could also be involved in pathological processes in the kidney.

## Introduction

Hypoxia has been implicated in the pathogenesis of various acute and chronic kidney diseases and plays a pivotal role in the final common pathway to end-stage renal disease (ESRD) (Nangaku, 2006; Tanaka et al., 2014; Pruijm et al., 2018; Packer, 2020). Hypoxia-induced factor (HIF) serves as the primary transcriptional regulator in hypoxia, although its role in the pathogenesis of renal diseases is not well understood. Functional HIF protein is a heterodimer comprised of α and β subunits, while 1α and 2α are the most ubiquitous α-subunits. Under normoxia, HIF1α and 2α undergo constant O_2_-dependent hydroxylation by prolyl-4-hydroxylases (PHDs), which are recognized by the Von Hippel–Lindau tumor-suppressor (VHL) E3 ligase complex and finally degraded by the proteasome (Schito and Rey, 2018). Under hypoxia, HIFα hydroxylation is obstructed, and then accumulated stable HIFα subunits migrate to the nucleus and combine with HIFβ. The HIF heterodimer binds to specific DNA sequences, namely, hypoxia-responsive elements (HREs), and modulates the expression of a series of genes mediating extensive adaptive hypoxic responses concerning angiogenesis, erythropoiesis, and cell survival, proliferation, and metabolism (Weidemann and Johnson, 2008).

However, in hypoxia-related renal pathology, the exact roles of HIF and HIF-induced reactions remain obscure. Moreover, discriminating HIF-regulated responses and hypoxic injuries is important, especially because PHD inhibitors (HIF stabilizers) have been used for renal anemia treatment. Oral PHD inhibitor roxadustat showed satisfactory efficacy and safety for renal anemia treatment (Chen et al., 2019a), whereas some studies suggest that HIF positively contributes to renal inflammation and fibrosis (Li et al., 2018, 2019). Thus, further recognizing HIF-related reactions in the kidney is important.

The kidney is one of the most energy-consuming organs, while tubular epithelial cells depend mostly on fatty acid oxidation (FAO) as their energy source (Kang et al., 2015). Disturbance of FAO underlies chronic renal fibrogenesis, which is partly attributed to ATP depletion (Kang et al., 2015). Excess local lipid accumulation was also associated with nephropathy progression (Abrass, 2004; Weinberg, 2006; Bobulescu, 2010). The mechanisms mainly include induction of mitochondrial dysfunction, endoplasmic reticulum stress, apoptosis, and reactive oxygen species and proinflammatory factor production (Weinberg, 2006; Bobulescu, 2010; Bhargava and Schnellmann, 2017). HIF was recently found to be linked with renal tubular lipid accumulation, although further investigations are needed to reveal the concrete mechanism (Schley et al., 2020).

Our study showed that HIF1α activation controls lipid utilization and composition in tubules through transcriptional regulation of metabolic genes regarding fatty acid mobilization, uptake, and utilization block. Such regulation might reflect a special pathway by which kidney tubules react to acute or chronic hypoxia, which could theoretically be involved in some pathological processes.

## Materials and Methods

### Cell Culture

The immortalized human renal proximal tubular cell line HK2 was cultured in DMEM/F12 (Gibco, Thermo Fisher Scientific, Waltham, MA, United States) supplemented with 10% fetal bovine serum (FBS, Gibco) and with 1% FBS in serum-limited medium. Cells were plated in 10-cm dishes or plates before exposure to 50 μM FG-4592 (HY-13426, MedChemExpress, dissolved in DMSO) for the indicated duration. All control groups were treated with the same dosage of DMSO. An airtight culture chamber containing 0.1% O_2_ and 5% CO_2_ was used for hypoxic cell culture.

### RNA-Seq Analysis

HK2 cells were cultured in DMEM/F12 with or without 50 μM FG-4592 treatment for 6 h and then harvested in TRIzol (15596026, Invitrogen, Thermo Fisher Scientific, Waltham, MA, United States). RNA samples were provided to the Vazyme Biotech Company for library construction and sequencing. The adaptor-trimmed and quality-filtered reads were mapped to hg19 using HISAT2. Transcript assembly was performed with StringTie. Fragments per kilobase of transcript sequence per million mapped fragments (FPKM) was used for expression level assessment.

### ChIP-Seq Analysis

Chromatin immunoprecipitation sequencing assays were performed as previously described (Hancock et al., 2019). Briefly, HK2 cells were treated under the same condition as the RNA-seq test. Cells were collected, and chromatin extract was sonicated for 15 s on and then 30 s off for 12 cycles. Immunoprecipitation was performed using anti-HIF1α (NB100-134, Novus Biologicals, Littleton, CO, United States). DNA was purified by a DNA Clean & Concentrator-5 kit (DCC-5, Zymo Research, Irvine, CA, United States). Libraries were constructed and sequenced on HiSeq 4000. The adaptor-trimmed and quality-filtered reads were aligned to hg19 using Bowtie2 with default parameters. Uniquely mapped reads were used for peak calling with MACS2. The RNA-seq and ChIP-seq datasets are available from the National Center for Biotechnology Information Gene Expression Omnibus.

### Lipidomics Profiling

Lipidomics profiling was performed in accordance with a previous study (Keerthisinghe et al., 2020). Briefly, the profiling of lipids was conducted using a reversed-phase HPLC system coupled to a 6550 Q-TOF system (Agilent, Santa Clara, CA, United States). The processing of metabolome data was carried out using the XCMS online web platform (Huan et al., 2017). Based on orthogonal partial least squares discriminant analysis (OPLS-DA), lipid metabolites that contributed to the lipidome difference according to the threshold of variable importance (VIP) value and fold changes were identified.

### Analysis of Lipolysis, FAO, TG Content, and ATP Production

Lipolysis was determined by measuring the free glycerol content in the extracellular medium with a colorimetric assay (F6428, Sigma, St. Louis, MO, United States) according to the manufacturers’ instructions (Maus et al., 2017). The results were represented as relative quantities. FAO, triglyceride (TG) content, and ATP production were tested with commercially available kits (ab222944, Abcam; A110-1-1, Nanjing Jiancheng, Nanjing, China; and S0026, Beyotime, Haimen, China, respectively) according to the manufacturers’ instructions. The results were normalized by protein concentration with the cells in each sample through a BCA assay.

### Immunofluorescence and Confocal Microscopy

Briefly, cells were cultured on coverslips and fixed with 4% PFA for 10 min, permeabilized with 0.1% Triton X-100 in PBS, and then incubated with anti-LAMP1 antibody (9091, CST, Danvers, MA, United States), followed by subsequent labeling with a secondary antibody. LDs were stained with 20 mg/ml BODIPY 493/503 (790389, Sigma) for 30 min. Fluorescence images of BODIPY 558/568 C_12_ absorption were collected using confocal microscopy (LSM 710, Zeiss, Jena, Germany).

### Mitophagy and Mitochondrial Membrane Potential Staining

Mitophagy detection was performed with a fluorescence probe (MD02, Dojindo, Rockville, MD, United States) according to the manufacturer’s protocol. In brief, the cells were stained for 1 h at 37°C/5% CO_2_ in the dark, and after being washed with PBS, fluorescence pictures were captured in living cells and analyzed using ImageJ 1.5 software. JC-10 (40707ES03, Yeasen, Shanghai, China) staining was carried out according to the manufacturer’s protocol. Fluorescence pictures were similarly captured in living cells, and the red-to-green fluorescence ratio was employed to evaluate the changes in mitochondrial membrane potential.

### Other Methods

Quantitative real-time PCR was performed after reverse transcription with PrimeScript^TM^ RT reagent kit (RR047A, Takara, Mountain View, CA, United States) with gene-specific primers and SYBR Green PCR Master Mix (43-676-59, Applied Biosystems, Foster City, CA, United States). Western immunoblotting was performed as described previously (Li et al., 2018). Anti-BNIP3 (3769, CST), anti-PLIN2 (15294-1-AP, Proteintech, Rosemont, IL, United States), anti-COX4 (11242-1-AP, Proteintech), and anti-GAPDH (HC301-01, Transgen, Strasbourg, France) were used. siRNA transfection was performed with Lipofectamine RNAiMAX (13778-075, Thermo Fisher) according to the manufacturer’s recommended protocol. Sequences of primers and siRNAs are provided in Supplementary Table 1.

### Statistical Analyses

All numerical results are shown as the mean±SEM. Two-tailed unpaired Student’s *t* test was used for statistical analysis, *n* = 3 for each group unless otherwise indicated.

## Results

### HIF1α Activation Suppressed Lipid Droplet Consumption in Tubular Cells

**FIGURE 1:**
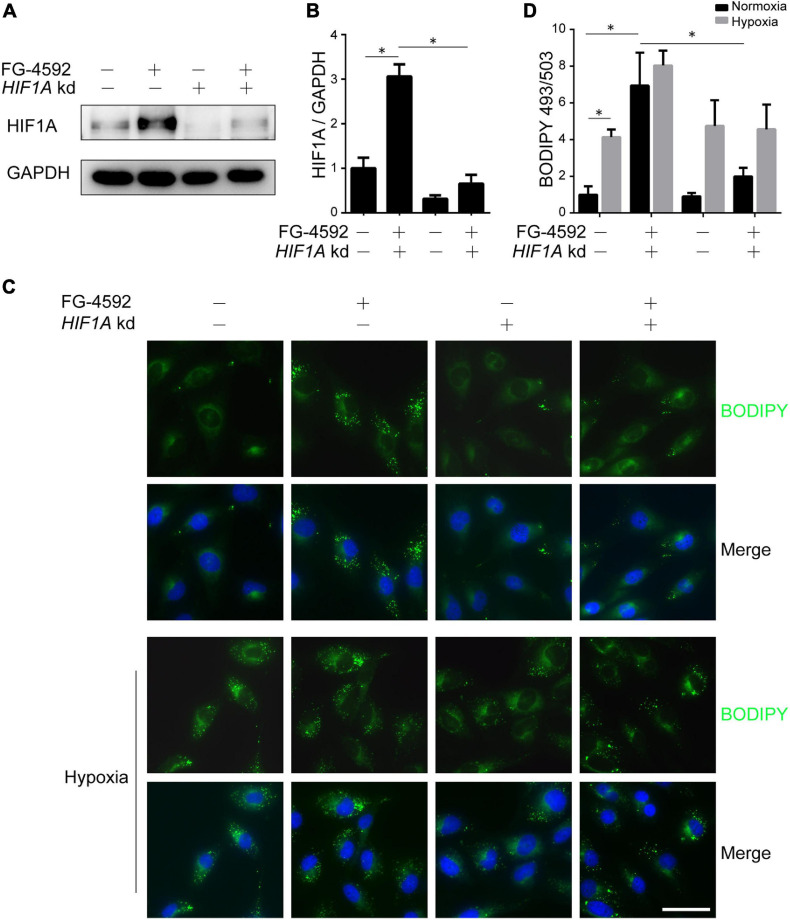
HIF1α activation leads to lipid accumulation in tubular cells. HK2 cells were treated or untreated with 50 μM FG-4592 in serum-limited medium for 24 h. Representative immunoblots **(A)** and quantitative analysis **(B)** of HIF1α in total cell lysates from HK2 cells. GAPDH was used as loading control. **(C,D)** Lipid droplets of HK2 cells were labeled by BODIPY 493/503 (green) staining, and the average areas of BODIPY 493/503 staining per cell were evaluated by optical density. Nuclei were stained with Hoechst 33258 (blue). Values were normalized to control group. *HIF1A* kd: *HIF1A* knockdown by siRNA. Scale bar: 50 μm. All images are representative of multiple experiments. Statistical analysis was performed using Student’s *t* test. *p < 0.05. Values are expressed as the mean ± SEM.

As HIFα proteins undergo constant hydroxylation and degradation in normoxia, to identify the direct effects of HIF activation on renal tubules, we used the PHD inhibitor FG-4592 to stabilize HIF protein. Consistent with a previous report (Li et al., 2019), PHD inhibition significantly increased the protein level of HIF1α (Figures 1A,B). HIF2α was also increased under FG-4592 treatment in cultured HK2 cells (Supplementary Figure 1A). We focused on the effects of HIF1α since HIF1α rather than HIF2α is predominantly expressed in tubular cells under hypoxia *in vivo* (Schödel et al., 2009) and is responsible for fibrogenesis in cultured tubular cells (Li et al., 2019). Efficient utilization of lipids was verified by BODIPY 493/503 stained LDs in HK2 cells cultured in serum-limited medium, which exhibited time-dependent depletion of LD content (Supplementary Figure 1B). We found that FG-4592 treatment repressed (though not fully blocked) lipid consumption and led to significant lipid accumulation, while *HIF1A* knockdown by siRNA reversed these effects (Figures 1C,D and Supplementary Figure 1B). These results suggested that HIF1α activation could increase lipid accumulation in tubular cells. Similarly, hypoxic conditions promoted cellular lipid accumulation, which could be slightly augmented by simultaneous FG-4592 treatment but could not be prevented by *HIF1A* knockdown, suggesting the coexistence of both active regulation and hypoxic restriction of lipid utilization (Figures 1C,D).

### Transcriptional Regulation of Metabolism-Related Genes by HIF1α

**FIGURE 2:**
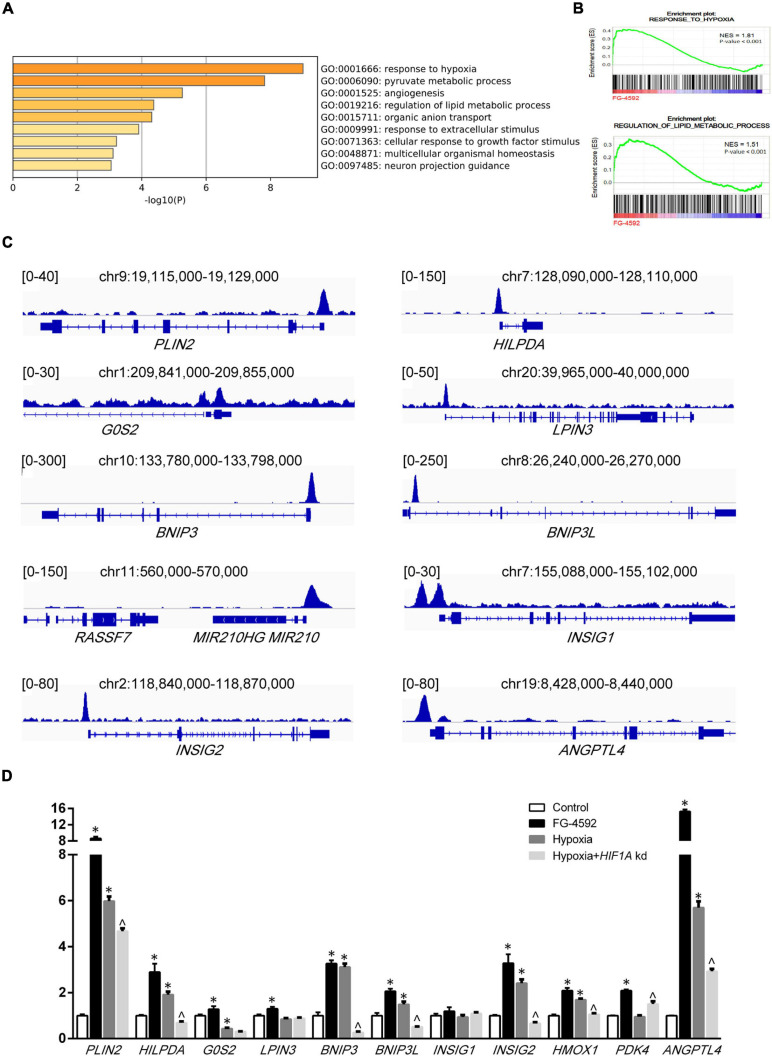
Direct transcriptional regulation by HIF1α. **(A)** Functional annotation clustering showed enriched Gene Ontology (GO) terms for biological processes among significantly differentially expressed genes in FG-4592-treated HK2 cells (6 h). **(B)** Enrichment plots from the GSEA performed for GO categories on transcriptomic profiling of HK2 cells treated with and without FG-4592. NES, normalized enrichment score. **(C)** Schematics of respective gene loci with HIF1α ChIP-seq peaks from HK2 cells under FG-4592 treatment. **(D)** Determination of gene expression levels in HK2 cells cultured in normoxia or in 6-h hypoxic (0.1% O_2_) conditions by RT-qPCR. FG-4592 was added simultaneously to groups indicated. *HIF1A* kd: *HIF1A* knockdown by siRNA. Statistical analysis was performed using Student’s *t* test. *p < 0.05 vs control groups; ^*p* < 0.05 vs hypoxia groups. Values are expressed as the mean ± SEM.

To further understand how HIF1α mediated metabolic changes in renal tubules, RNA sequencing (RNA-seq) was performed to examine gene expression under FG-4592 treatment. A total of 193 significantly differentially expressed genes (DEGs, | log_2_ fold change| >1, *p* < 0.05) were identified (Supplementary Figure 1C). We next examined the function of these DEGs. As shown in Figure 2A, the top four enriched GO terms for biological processes were response to hypoxia (GO:0001666), pyruvate metabolic process (GO:0006090), angiogenesis (GO: 0001525), and regulation of lipid metabolic process (GO: 0019216). In addition, a gene set enrichment analysis (GSEA) of the total transcriptome also showed that cells treated with FG-4592 displayed enrichment of the gene expression signature that corresponds to the hypoxic response and lipid metabolism regulation category (*p* < 0.001; FDR < 0.001; Figure 2B).

We examined the functions of upregulated genes that have relationships with lipid metabolism or mitochondrial function (Table 1). To further investigate the association between gene upregulation and HIF1α, we experimentally constructed the chromatin immunoprecipitation sequencing (ChIP-seq) of HIF1α. We found that HIF1α directly binds to the promoters of many of these genes (Figure 2C). Upregulation of most of the genes mentioned above could be verified by qPCR under FG-4592 treatment and under bona fide hypoxic conditions (Figure 2D). *HIF1A* knockdown under hypoxic conditions attenuated the upregulation of some of these genes (*p* < 0.05). These findings suggested that HIF1α directly binds to the promoters of metabolic genes and upregulates their expression.

**TABLE 1:** FG-4592-upregulated genes that relate to lipid metabolism or mitochondrial function.

**Gene symbol**	**Fold change (FPKM)**	**HIF1α promoter binding (±)**	**Functions relate to lipid metabolism or mitochondria**
PLIN2	5.76	+	Inhibition of ATGL and lipophagy (Kaushik and Cuervo, 2015)
HILPDA	4.39	+	Inhibition of ATGL (Padmanabha Das et al., 2018)
G0S2	2.84	+	Inhibition of ATGL (Cerk et al., 2018)
LPIN3	1.82	+	Diacylglycerol synthesize (Donkor et al., 2007)
HMOX1	3.19	−	Mitochondrial membrane damage (Zukor et al., 2009); mitophagy (Meyer et al., 2018)
PDK4	2.39	−	Mitochondrial respiration impairment (Oh et al., 2017; Ma et al., 2020); Δψm decrease (Oh et al., 2017)
BNIP3	3.48	+	Mitophagy; mitochondrial respiration impairment (Rikka et al., 2011); mitochondrial permeability transition and Δψm decrease (Zhang et al., 2009; Liu et al., 2017b)
BNIP3L	3.31	+	Mitophagy; mitochondrial fission, Δψm decrease (da Silva Rosa et al., 2020); regulates mitochondrial permeability transition and calcium homeostasis (Mughal et al., 2018)
MIR210	1.33	+	Mitochondrial inner membrane (ETC iron-sulfur clusters) and respiration impairment (Chan et al., 2009; Nakada et al., 2020)
MIR210HG	2.72	+	
INSIG1	1.59	+	Inhibition of SREBP/LDLR (Shimano and Sato, 2017)
INSIG2	3.68	+	Inhibition of SREBP/LDLR (Shimano and Sato, 2017)
ANGPTL4	15.76	+	Inhibition of LPL-mediated hydrolysis of plasma TG to FA and subsequent uptake (Aryal et al., 2019); Promote lipoproteins and FA uptake (Bruce et al., 2018; Loving et al., 2021)

### HIF1α Decreased FA Availability

**FIGURE 3:**
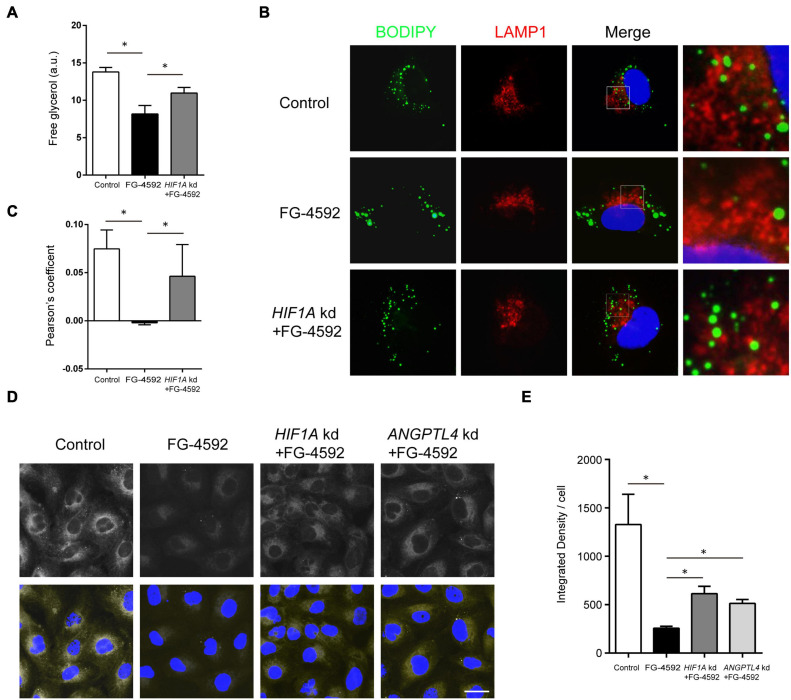
HIF1α decreased FA availability by inhibiting lipolysis and FA uptake. **(A)** Free glycerol released by HK2 cells after 24 h of culture in serum-limited medium in the presence or absence of 50 μM FG-4592. Values were normalized to protein concentration. a.u, arbitrary units. **(B,C)** Colocalization of LDs and lysosomes was assessed by the Pearson’s coefficient. HK2 cells were cultured for 24 h with or without FG-4592 and then in serum-limited culture for 4 h. Lysosomes were marked by immunofluorescence staining of LAMP1 (red). LDs were labeled by BODIPY 493/503 staining. Blue: DAPI. **(D,E)** HK2 cells were cultured in the presence or absence of 50 μM FG-4592 for 6 h and then treated with 2 μM Red C_12_ for 2 h. Red C_12_ uptake was evaluated by confocal microscopy. Scale bar: 20 μm. All images are representative of multiple experiments. *HIF1A* and *ANGPTL4* kd: gene knockdown by siRNA. *p < 0.05. Values are expressed as the mean ± SEM.

Among significantly upregulated lipid metabolism genes, some were closely linked to lipid hydrolysis (lipolysis) inhibition. In addition to the process in cytoplasm, lipolysis of the LD portion in lysosomes, named lipophagy, is another important method of lipid catabolism (Zechner et al., 2017). PLIN2, HILPDA, and G0S2 are known to inhibit the activity of ATGL, the key cytoplasmic lipolysis enzyme that also regulates lipophagy by interacting with LC3, while PLIN2 can directly impede lipophagy (Kaushik and Cuervo, 2015; Martinez-Lopez et al., 2016; Cerk et al., 2018; Padmanabha Das et al., 2018). FG-4592 upregulated *PLIN2*, *HILPDA*, *G0S2*, and *LPIN3* (catalyzing diacylglycerol synthesis) expression to approximately 5.8-, 4.5-, 2.9-, and 1.8-fold, respectively (FPKM). Therefore, we hypothesized that inhibition of lipolysis and lipophagy might contribute to lipid accumulation. As expected, FG-4592 treatment inhibited lipolysis levels, while knockdown of *HIF1A* reversed this inhibition (Figure 3A). Immunofluorescent staining of the lysosome marker LAMP1 and LD staining with BODIPY 493/503 were carried out. Some LDs were distributed in close proximity to lysosomes, which were recognized as LDs engaging in lipophagy (Schulze et al., 2020). FG-4592 treatment decreased the portion of LDs in the vicinity of lysosomes, while *HIF1A* knockdown restrained this trend (Figures 3B,C). These results indicated that HIF1α inhibited lipolysis, and especially lipophagy.

Extracellular uptake is another essential source of FA in tubules (Meyer et al., 1997). We then evaluated FA uptake with BODIPY 558/568 C_12_ (Red C_12_), a saturated FA analog with a tail composed of 12 carbons and a BODIPY 558/568 fluorophore (Minami et al., 2017). The results showed that fluorescent intensities in the FG-4592 groups were obviously less than those in the controls, indicating decreased Red C_12_ absorption, while simultaneous *HIF1A* knockdown increased the fluorescent intensities to some degree, thus verifying the role of HIF1α in limiting FA uptake (Figures 3D,E).

Several upregulated genes are associated with lipoprotein uptake: *ANGPTL4*, *INSIG2*, and *INSIG1*. ANGPTL4 is a potent inhibiting factor of lipoprotein lipase (LPL) (Aryal et al., 2019) that directly promotes lipoprotein and FA uptake and hydrolyzes TG in circulating lipoproteins (Bruce et al., 2018; Loving et al., 2021). *ANGPTL4* expression was upregulated approximately 15.8-fold (FPKM) by FG-4592. We conducted siRNA knockdown of *ANGPTL4* and proved its role in partial remission of FA uptake inhibition caused by FG-4592 (Figures 3D,E).

### HIF1α Activation Decreased FA β-Oxidation

**FIGURE 4:**
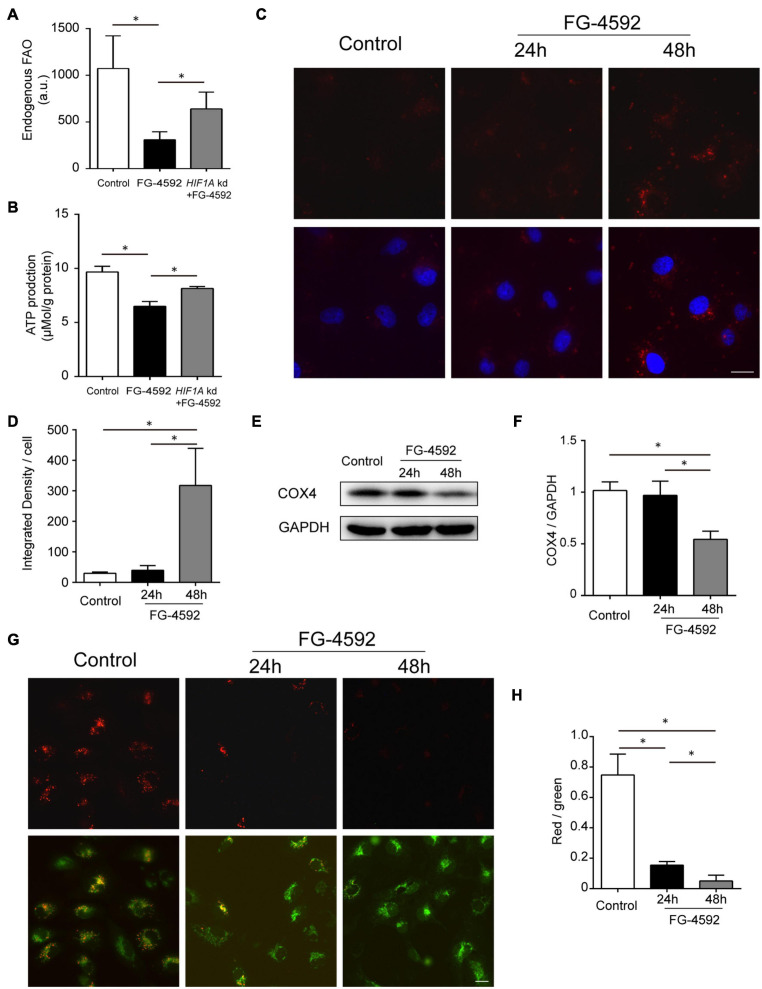
HIF1 activation decreased FA β-oxidation. **(A,B)** Endogenous FAO activity assay and ATP production assay of HK2 cells cultured with or without 24 h treatment of FG-4592 (50 μM). *HIF1A* kd: Cells with *HIF1A* siRNA knockdown before treatment. **(C,D)** Assessment of mitophagy in HK2 cells with a mitophagy detection reagent. The fluorescence intensity of the dye (red) was quantified. Nuclei were stained with Hoechst 33258 (blue). **(E,F)** Representative immunoblots and semi-quantification of COX4 in total cell lysates from HK2 cells. GAPDH was used as loading control. **(G,H)** Mitochondrial membrane potential (Δψm) changes were detected by JC-10 staining and captured by fluorescence microscopy. Then, the red-to-green fluorescence ratio was used to quantify mitochondrial potential. Scale bars: 20 μm. Values are expressed as the mean ± SEM (*p < 0.05).

Hypoxia-induced factor has been reported to suppress mitochondrial FAO in cancer cells *via* acyl-CoA dehydrogenase MCAD and LCAD regulation (Huang et al., 2014). We then tested the relationship between HIF1 activation and FAO in HK2 cells and found decreased FAO activity caused by FG-4592 (Figure 4A). ATP production was similarly regulated by HIF1α at 24 h (Figure 4B). As previously mentioned, our results indicated that HIF activation significantly increased *BNIP3* and *BNIP3L* expression, which are key factors that provoke mitophagy (Figure 2C and Table 1). We tested whether mitophagy was induced by HIF activation in HK2 cells with a probe detecting mitochondria-lysosome fusion. As shown in Figures 4C,D, enhanced mitophagy was identified after 48 h of FG-4592 treatment but not at 24 h. In addition, mitophagy was evaluated by measuring the protein level of COX IV (reflecting mitochondrial mass), which also implicated mitochondrial reduction after 48 h of treatment rather than 24 h (Figures 4E,F). These results suggest that other mechanisms are involved in inhibiting mitochondrial oxidation before mitophagy-related mitochondrial loss.

Several upregulated genes have been shown to restrain basal mitochondrial respiratory capacity with respective mechanisms other than mitophagy, including *BNIP3* (Zhang et al., 2009; Rikka et al., 2011; Liu et al., 2017b), *BNIP3L* (Mughal et al., 2018; da Silva Rosa et al., 2020), *HMOX1* (Zukor et al., 2009; Meyer et al., 2018), *PDK4* (Oh et al., 2017; Ma et al., 2020), and *MIR210* (microRNA-210) (Chan et al., 2009; Nakada et al., 2020; Table 1). The mitochondrial membrane potential (Δψm) normally remains stable for the respiratory chain to generate ATP and thus can be used as a fundamental indicator of mitochondrial function (Zorova et al., 2018; Li et al., 2020). We found that Δψm was reduced after 24 h of FG-4592 treatment, which was further exacerbated after 48 h (Figures 4G,H), suggesting that mitochondrial function was compromised by activation of HIF and downstream factors before substantial mitophagy induction.

### Sustained High Expression of HIF1α-Related Metabolic Genes After Acute Kidney Injury

**FIGURE 5:**
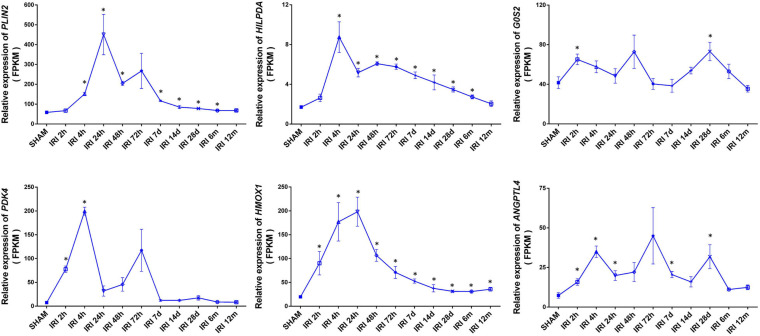
Elevated expression of lipid metabolism genes at the whole-kidney level after acute kidney injury (AKI). Relative expression of the indicated genes is shown by FPKM. Data came from published RNA-seq profiles (GEO accession GSE98622). IRI: ischemia/reperfusion injury. SHAM: sham IRI surgery controls. *X*-axis ticks show different examination time points following IRI of respective groups. Values are expressed as the mean ± SEM (*p < 0.05).

Acute ischemia is a common cause of acute kidney injury (AKI). To determine whether HIF1α upregulated metabolic genes participate in AKI, we studied published RNA-seq profiles (GEO accession GSE98622) of murine kidneys taken at different times after 21 min of warm ischemia (Liu et al., 2017a). Several HIF1α-related metabolic genes were highly expressed at the whole-kidney level after ischemia reperfusion. Unexpectedly, these genes showed sustained high expression for up to weeks or months, suggesting the involvement of HIF-mediated metabolic alterations in the chronic tissue remodeling process after AKI (Figure 5). The expression level generally peaked at 4–24 h and declined over time, maintaining a mild rise for a relatively long period. The expression of HMOX1 showed an approximately 1.8-fold increase even at 12 months (*p* < 0.05).

### HIF1α-Induced Lipidomics Alterations in Kidney Tubular Cells

**FIGURE 6:**
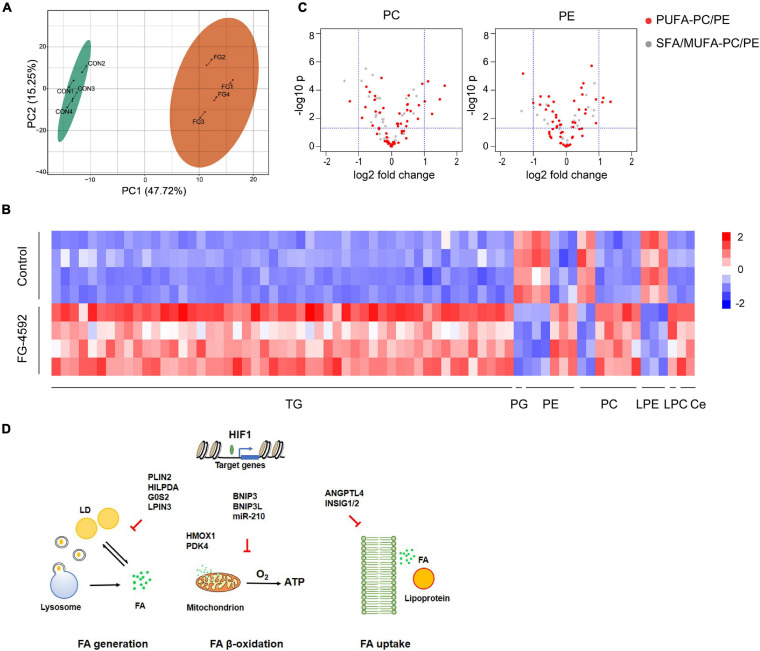
HIF1α-induced lipidomics alterations in kidney tubular cells. **(A)** PCA score plots based on the identified lipid metabolites by LC-MS/MS of HK2 cells treated or untreated with 50 μM FG-4592. CON: Control group. FG: FG-4592 treated group. *N* = 4. **(B)** Heatmap of the differentially enriched lipid species. | log_2_ fold change| > 1, VIP ≥ 1. TG, triglyceride; PG, phosphatidylglycerol. PE, phosphatidylethanolamine; PC, phosphatidylcholine; LPE, lysophosphatidylethanolamine; LPC, lysophosphatidylcholine; Ce, ceramide. **(C)** Volcano plots showing the changes in PC/PEs grouped as PUFA-PCs/PEs (red fill) and saturated/monounsaturated fatty acyl (SFA/MUFA)-PCs/PEs (white fill) between FG-4592 treated and control groups. **(D)** Schematic diagram displaying HIF1 activation-regulated reprograming of FA metabolism.

In addition to energy storage, different cellular lipid contents have essential roles in basic cell physiology, functioning as membrane structure components or cell signaling molecules. Profound changes in the renal lipidome may exert either protective or toxic effects in various renal injuries (Rao et al., 2016). To further investigate lipid metabolic transformation triggered by HIF activation, we studied the lipidome with LC-MS/MS. Treatment with FG-4592 led to distinct lipidomic compositions (Figure 6A). A total of 402 individual lipid species were detected, among which 62 lipids were significantly upregulated and 9 were downregulated (| log_2_ fold change| >1, VIP ≥ 1) (Figure 6B). The majority of upregulated lipid species were TGs, which was in accordance with the intracellular LD accumulation phenomenon and HIF mediated lipid catabolism inhibition mentioned above.

Eukaryotic membranes are mainly composed of glycerophospholipids (GPLs), sphingolipids, and sterols. Phosphatidylcholine (PC), phosphatidylethanolamine (PE), phosphatidylserine (PS), and phosphatidylinositol (PI) are the major classes of membrane GPLs, while PC and PE account for 41–57 mol% and 17–38 mol% of the total phospholipids, respectively (Yang et al., 2018). We identified differentially enriched GPLs and found that the upregulated GPLs were particularly PCs and PEs that contain polyunsaturated fatty acyl (PUFA) chains, such as arachidonic acid (C20:4) (Figure 6C). Increased lipid unsaturation might correlate with HILPDA activation as HIF1α/2α was shown to significantly enrich polyunsaturated lipids *via* HILPDA in clear-cell carcinomas, independent of its ATGL-suppressing function (Zou et al., 2019).

We identified an increase in two sphingolipids: ceramide(m18:1/22:1) and ceramide(m18:1/20:0). Ceramide was demonstrated to regulate various cellular responses like inflammation and apoptosis and was linked closely with renal dysfunction (Zhu and Scherer, 2017; Huwiler and Pfeilschifter, 2018). HIF has been proven to regulate ceramide metabolism *via* NEU3 (Xie et al., 2017) or ACER2 (Zhang et al., 2019) in different tissues, but not in our RNA-seq data; thus, additional studies are needed to clarify the regulatory mechanism.

Notably, one phosphatidylglycerol (PG), which is the precursor for cardiolipin synthesis, was downregulated in the FG-4592-treated groups. Cardiolipin is almost exclusively found in the mitochondria (Martinou and Youle, 2011); thus, it may reflect downregulated mitochondrial biogenesis. Three lysophosphatidylethanolamines (LPEs), which are derived from the hydrolytic cleavage of fatty acid residues at the sn-2 position of PEs, were downregulated, suggesting decreased membrane lipid catabolism. However, in the transcriptome results, PG metabolism-related genes CDS1/2, PGS1, and CRLS1 and LPE-related phospholipase genes were not differentially expressed between groups, suggesting possible involvement of other mechanisms apart from HIF-associated transcription regulation.

## Discussion

The possible correlation between kidney dysfunction and lipid metabolism dysregulation has been studied for decades. Renal tubules, especially proximal tubules, are in high demand for ATP to maintain constant reabsorption activities that are crucial for body fluid and electrolyte balance. As β-oxidation of FA is the principal way tubules obtain ATP, the homeostasis of lipid metabolism has particular importance to renal physiology (Kang et al., 2015). In this study, we tried to further investigate the associations between hypoxic responses and tubular lipid metabolism and found that HIF1α inhibited tubular FA metabolism in a robust manner, involving multiple genes (Figure 6D).

Renal lipid accumulation is seen in systematic dyslipidemia, such as nephrotic syndrome or diabetic nephropathy (Falkevall et al., 2017; Agrawal et al., 2018). Metabolic disorders of intrinsic kidney cells also underlie lipid accumulation and lipotoxicity, such as in injured tubular epithelial cells (Zager et al., 2005; Tran et al., 2016). The mechanism of lipid accumulation-associated kidney detriment is not quite clear. Several studies have suggested that lipotoxicity in the kidney is ascribed to mitochondrial, lysosomal, or endoplasmic reticulum stresses, accompanied by resultant inflammation and fibrosis (Declèves et al., 2014; Szeto et al., 2016; Yamamoto et al., 2017). Additionally, disturbed lipid metabolism *per se* might jeopardize kidney function by hindering energy supply. Notably, Kang et al. disclosed that defective tubular FAO plays a key role in renal fibrogenesis, whereas accumulation of TG and long-chain fatty acids alone was not sufficient to cause renal fibrosis (Kang et al., 2015). In AKI animal models, accumulated kidney lipids went with downregulated FAO-related factors, while activation of PPARα (Li et al., 2005, 2009; Nagothu et al., 2005), PGC1α (Tran et al., 2011, 2016), and CPT1 (Idrovo et al., 2012) provided renal protective effects. By analyzing public data, we found sustained high expression of several genes upregulated by HIF stabilization and may affect lipid metabolism, which have been seldom studied in this issue.

Metabolic reprograming is a basic event in hypoxic responses. HIF is a potent metabolic regulator that functions at both the tissue and cellular levels, reprograming energy production and utilization to cope with a low oxygen supply (Schito and Rey, 2018). Other than the well-known effect of enhancing anaerobic glycolysis, studies have illustrated that HIF promotes lipid storage in liver and clear cell renal cell carcinoma (Rankin et al., 2009; Qiu et al., 2015; Du et al., 2017). Very recently, PHD inhibition was found to increase lipid accumulation in human tubular epithelial cells, suggesting participation of HIF in tubular lipid regulation, although the concrete mechanism still needs further investigation (Schley et al., 2020). We provide evidence that HIF1α activation has inhibitory effects on tubular lipid metabolism, including FAO, leading to lipid accumulation. Consistent with our results, another recent study showed that the PHD inhibitor enarodustat can counteract diabetes-induced upregulation of fatty acid and amino acid metabolism genes and thus may have protective roles against diabetic kidney disease progression (Hasegawa et al., 2020).

In the current study, HIF1α suppressed lipid utilization at both the hydrolysis and mitochondrial oxidation levels, reducing ATP production. Lipolysis is carried out by cytosolic lipases or lysosomal lipases. The latter, called lipophagy, has unique importance in maintaining energy homeostasis of the proximal tubules during prolonged starvation (Minami et al., 2017). We showed that lipolysis, especially lipophagy, was suppressed by HIF1α. LD is composed of a neutral lipid core and a surrounding phospholipid monolayer with functional proteins such as PLIN2. It dynamically regulates FA utilization and neutral lipid storage to avoid excessive cytoplasmic FA induced toxicity (Farese and Walther, 2009). We showed that the FA supply from LDs and the extracellular space was reduced after HIF1α activation, while mitochondrial FAO was also decreased. FAO downregulation was not the result of limited FA availability because the lipidome showed that the cellular FA level remained stable instead of exhausted.

The mitophagy-related genes *BNIP3* and *BNIP3L* were significantly upregulated, and augmented mitophagy was verified by fluorescence probe and reduced mitochondrial mass at 48 h under FG-4592 treatment; however, repressed FAO was observed before that time point. Several upregulated genes were reported to be linked with mitochondrial respiration perturbation by inducing a number of functional changes: mitochondrial permeability transition and/or Δψm loss (Zhang et al., 2009; Liu et al., 2017b; Oh et al., 2017; Meyer et al., 2018; Mughal et al., 2018; da Silva Rosa et al., 2020); mitochondrial fission (da Silva Rosa et al., 2020); mitochondria-endoplasmic reticulum association disruption (Ma et al., 2020); and mitochondrial inner structure disorganization (Zukor et al., 2009; Nakada et al., 2020). Further studies are needed to illustrate the mechanisms involved in HIF1-suppressed β-oxidation, which might be a multiple factor-involved process.

Canonical factors known to mediate FA uptake, such as CD36, FATPs, and FABPs, were not significantly changed by the FG-4592 treatment according to our results. Remarkably, ANGPTL4 was upregulated by HIF1α and showed sustained elevation in the kidneys after AKI. In the skeletal muscle and heart, ANGPTL4 potently inhibits cell surface LPL activity, which mediates hydrolysis of plasma TG to FA for tissue uptake (Clement et al., 2014; Aryal et al., 2019). Recently, it was found that LPL directly contributes to lipoprotein and lipid (including FA) import (Bruce et al., 2018; Loving et al., 2021), while our study showed that downregulated Red C_12_ levels in FG-4592 treated cells could partly be attributed to the HIF1/ANGPTL4 pathway. INSIG1/2 functions to inhibit LDL absorption by suppressing the SREBP/LDLR pathway (Shimano and Sato, 2017). Whether HIF1α-upregulated ANGPTL4 and INSIG1/2 affect lipoprotein uptake or hydrolysis in the kidney still needs further investigation.

The role of bioactive sphingolipids in the adverse effects of lipid accumulation and dyslipidemia has recently gained increasing attention (Hannun and Obeid, 2018). As the central molecule in sphingolipid metabolism, ceramide has been illustrated to be proapoptotic, proinflammatory and profibrotic and involved in the pathogenesis of multiple disorders (Summers et al., 2019). According to the lipidome study, we identified two types of significantly accumulated ceramides after HIF activation. Ceramide storage and turnover mainly take place in LDs (Yang et al., 2018), although it is not clear whether its increase is associated with LD accumulation. We also identified increased PCs and PEs with PUFA chains, which might be more vulnerable to peroxidation reactions (Bailey et al., 2015), pending further confirmation.

Our study proposes the following question: would PHD inhibitors or HIF activation harm kidney function by FAO inhibition? HIF-PHD inhibitors have been explored in clinical trials of renal anemia, as HIF modulates erythropoietin production. FG-4592, also named roxadustat, has been approved for the treatment of anemia in patients with CKD in China (Voit and Sankaran, 2020). Oral roxadustat was effective for anemia in Chinese patients undergoing or not receiving dialysis, without an additional risk of renal impairment during treatment (Chen et al., 2019a,b). Nonetheless, adverse effects of HIF activation in the kidneys should not be simply ignored according to the results of clinical trials in selected patients. A number of studies have verified the link between HIF1α and tubulointerstitial inflammation or fibrosis (Higgins et al., 2007; Kimura et al., 2008; Luo et al., 2014; Kraus et al., 2018; Li et al., 2018; Chen et al., 2020). On the other hand, oral administration of HIF-PHD inhibitors showed systematic effects out of the kidney, such as benefiting cholesterol metabolism, which may subsequently affect kidney metabolism (Chen et al., 2019b). Some researchers have suggested that the balance of beneficial or deleterious effects of HIF-PHD inhibitors in injured kidney depends on the timing of administration (Yu et al., 2012). The dosage adopted should also be important for PHD inhibitor effects. Actually, the effective FG-4592 concentration in anemia treatment was much lower than that in our experiment (Groenendaal-van de Meent et al., 2021).

Our study further illustrated how HIF functions in tubular lipid metabolism regulation. Reduced lipolysis, FA uptake, and FAO combined to reprogram tubular lipid utilization from a high-energy production state to relatively low efficiency. This reprograming may keep cells away from FA-associated cytotoxicity and excessive mitochondrial ROS production in acute hypoxia but would increase overall lipid accumulation. In summary, HIF1α activation reorganized lipid homeostasis in renal tubules by blocking FA utilization at different levels, which reduced ATP availability and further led to lipid accumulation. Mitigating in such changes in hypoxia-related renal disorders may provide novel approaches for treatment.

## Data Availability Statement

The data presented in the study are deposited in the Gene Expression Omnibus repository https://www.ncbi.nlm.nih.gov/geo/, accession number GSE175681.

## Author Contributions

JY and WL contributed to the conception of the study. WL and AD performed the experiment. YX contributed significantly to analysis. LX helped perform the analysis. All authors contributed to the article and approved the submitted version.

## Conflict of Interest

The authors declare that the research was conducted in the absence of any commercial or financial relationships that could be construed as a potential conflict of interest.
