# Supplementary figures and images for: Transcription-Based Multidimensional Regulation of Fatty Acid Metabolism by HIF1α in Renal Tubules
Source: Front Cell Dev Biol. 2021 Jul 2;9:690079. doi: 10.3389/fcell.2021.690079 (PMC8283824; doi:10.3389/fcell.2021.690079)

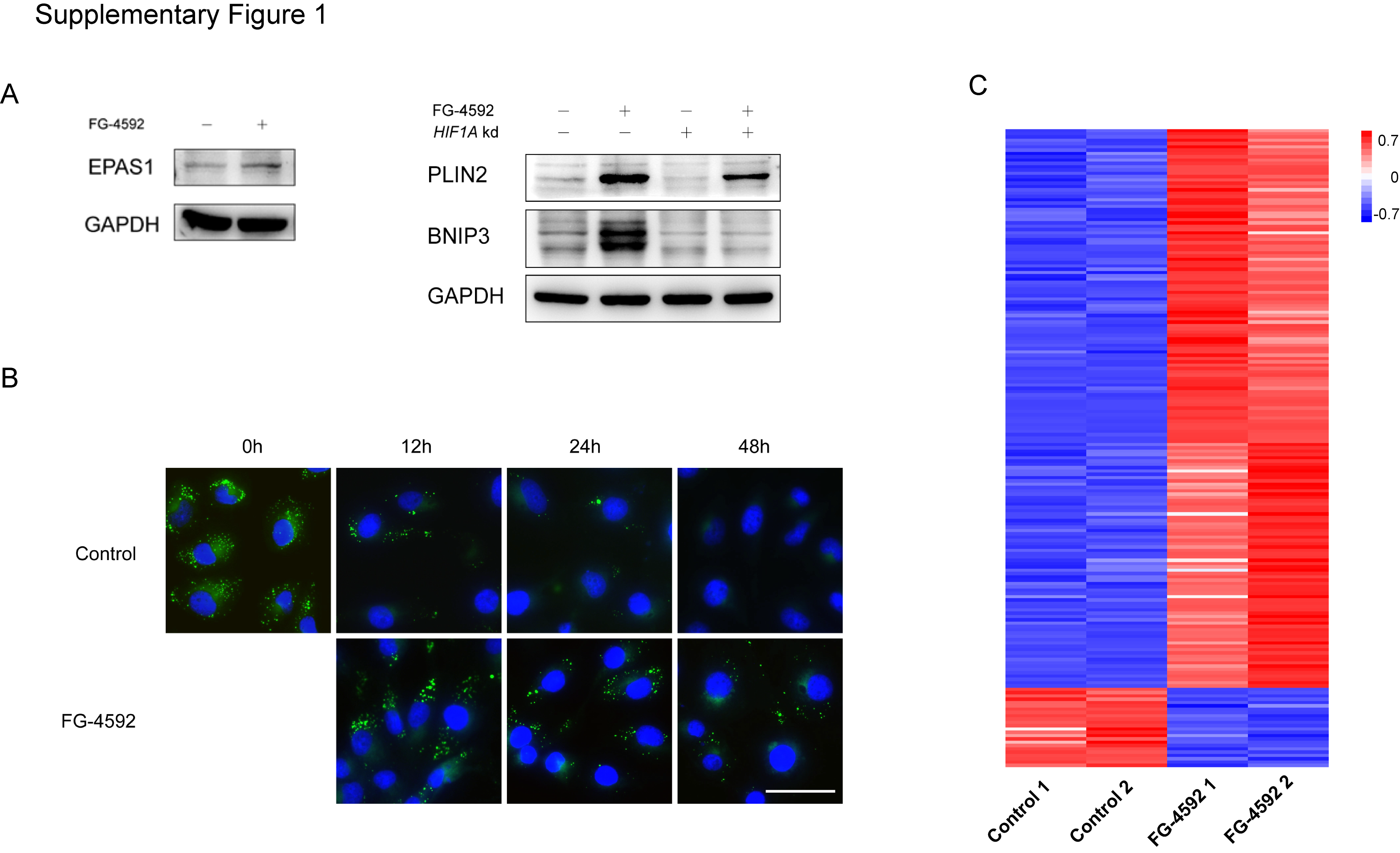

Supplement: Supplementary file 2 [file Image_1.TIF]
